# Enhancing energy expenditure and enjoyment of exercise during pregnancy through the addition of brief higher intensity intervals to traditional continuous moderate intensity cycling

**DOI:** 10.1186/s12884-016-0947-3

**Published:** 2016-07-15

**Authors:** Ming Jing Ong, Karen E. Wallman, Paul A. Fournier, John P. Newnham, Kym J. Guelfi

**Affiliations:** School of Sport Science, Exercise and Health, The University of Western Australia, 35 Stirling Highway, Crawley, WA 6009 Australia; School of Women’s and Infants’ Health, The University of Western Australia, Perth, WA Australia

**Keywords:** Gestation, Exercise training, Maternal heart rate

## Abstract

**Background:**

Current guidelines recommend that pregnant women without contraindications should engage in 30 min or more of moderate intensity exercise on most days of the week, however, many women fail to achieve this goal. This study examined the effect of adding brief higher intensity intervals to traditional continuous moderate intensity exercise on energy expenditure and the enjoyment of exercise in late pregnancy. This is important to determine given that any additional energy expenditure resulting from higher intensity intervals may be meaningless if enjoyment is compromised, since long-term adherence will likely be low.

**Methods:**

In this study, 12 healthy pregnant women at 30 ± 1 weeks gestation, aged 35 ± 6 years with a BMI of 27.1 ± 4.3 kg/m^2^ performed either 30 min of continuous cycling exercise (CONT) at a steady power output equivalent to 65 % age-predicted heart rate maximum or an equivalent period of interval cycling (INTV) consisting of continuous cycling at the same power output as CONT, but with the addition of six 15-s self-paced higher intensity efforts throughout, performed at regular intervals, on separate occasions in a counterbalanced order.

**Results:**

Mean cycling power output, heart rate, oxygen consumption and energy expenditure were higher during INTV compared with CONT (*P* < 0.05). However, there was no difference in mean rate of perceived exertion between conditions. Enjoyment of exercise was higher with INTV (*P* = 0.01).

**Conclusions:**

The addition of six 15-s higher intensity intervals to continuous moderate intensity exercise effectively increased energy expenditure by 28 %, at the same time as enhancing the enjoyment of exercise in late pregnancy. While these findings may be specific to recreationally active women, this study provides a rationale for future studies to examine the physiological and psychological responses to regular interval training during pregnancy to optimise exercise prescription.

**Trial registration:**

Australian New Zealand Clinical Trials Registry ACTRN12616000680460. 25 May 2016 (Registered retrospectively).

## Background

Stationary cycling is a recommended mode of exercise for pregnant women without obstetric complications [1] and may be preferable to walking during late gestation as it facilitates a higher self-paced intensity of exercise [2]. This in turn allows for greater energy expenditure and an augmented decline in postprandial glucose concentration for a given duration of exercise, without compromising the enjoyment of exercise [2]. A program of regular home-based stationary cycling also appears to have favourable effects on maternal fitness and glucose tolerance in previously inactive obese pregnant women [3], and may assist with the management of daily postprandial blood glucose concentrations in women diagnosed with gestational diabetes mellitus [4]. However, the specific format and intensity of stationary cycling to safely optimise health and fitness benefits, together with enjoyment for the pregnant woman is not known.

Current guidelines recommend that pregnant women without medical and obstetric complications should engage in 30 min or more of moderate intensity exercise on most days of the week [1]. However, with multiple barriers to exercise for busy mothers [5], more than 50 % of pregnant women do not achieve this [6, 7]. It has recently been suggested that vigorous intensity exercise may also be an important goal for some pregnant women [8–10], but engaging in this level of exercise intensity may require more regular prenatal monitoring for maternal and fetal wellbeing [11]. In addition, vigorous intensity exercise may not be ideal for previously sedentary women. Incidentally, interval exercise is a popular way to incorporate some vigorous exercise into an exercise routine as it involves alternating periods of higher and lower intensity exercise over time. This allows for a higher intensity training stimulus with partial recovery between efforts. Indeed, in a non-pregnant population interval training has been reported to provide superior health benefits compared with moderate intensity continuous exercise [12–14] and to enhance exercise enjoyment and adherence [15]. The addition of brief higher intensity intervals to traditional continuous moderate intensity training may also provide an opportunity to safely optimise health and fitness benefits for the pregnant woman by increasing the energy expenditure of an exercise session, at the same time as maximising enjoyment. The latter is important given that the enjoyment of exercise is an important predictor of exercise adherence [16]. Meanwhile, strategies to increase overall energy expenditure during exercise may assist pregnant women to meet weekly volume-based targets with fewer exercise sessions or a reduced overall duration of exercise. Alternatively, increasing the overall weekly energy expenditure from exercise likely has benefits for the prevention of gestational diabetes, pre-eclampsia and excessive weight gain [17]. In addition to these pregnancy-specific benefits, increasing energy expenditure through regular exercise has well-established benefits for reducing life-long all-cause mortality, risk of cardiovascular disease, type 2 diabetes and some cancers [18, 19].

Previous research has shown that a 6-week program of stationary cycling including brief intervals (consisting of 15 to 60 s at 75–85 % age-predicted HR_max_ performed every 2 min, interspersed with lower intensity recovery at 55–65 % age-predicted HR_max_ between efforts) commenced at 28–29 weeks of pregnancy is well-tolerated, with excellent program compliance (96 % of scheduled sessions completed) [20]. Furthermore, this program was effective for improving daily postprandial glucose control, maternal fitness, and attitudes and intentions towards exercise, with no detrimental effects on obstetric outcomes [20]. Whether similar health and fitness benefits may have been obtained with traditional moderate intensity exercise alone is not clear. Regardless, the first step towards addressing the issue of the optimal format and intensity of stationary cycling to safely optimise health and fitness benefits, together with enjoyment for the pregnant woman is to examine how the addition of brief self-paced higher intensity intervals to continuous exercise affects energy expenditure and the enjoyment of exercise in pregnancy. Understanding the acute responses to a single bout of exercise will provide justification for longer-term interventions of this nature in the future. Therefore, the aim of the present study was to investigate the effect of adding brief higher intensity intervals to traditional moderate-intensity continuous cycling on energy expenditure and the enjoyment of exercise in late pregnancy. Although higher intensity interval exercise that is performed for the same duration as continuous moderate-intensity exercise should result in greater energy expenditure, it is not known whether this would compromise exercise enjoyment in pregnancy. We hypothesise that the addition of brief higher intensity intervals to moderate intensity continuous cycling will increase the overall energy expenditure and intensity of exercise, at the same time as enhancing the enjoyment of exercise.

## Methods

### Participants

Healthy, non-smoking women (*N* = 12) with uncomplicated singleton pregnancies were recruited upon entering their third trimester at 30 ± 1 weeks of gestation. Participants were 35 ± 6 years of age with pre-pregnancy body mass index (BMI) of 23.3 ± 3.0 kg/m^2^ and BMI of 27.1 ± 4.3 kg/m^2^ at the familiarisation session. Five of the participants were experiencing their first pregnancy, while the remaining seven already had ≥ one child. Pre-pregnancy exercise participation for all women was ≥ 1 session per week of at least 30 min of moderate intensity exercise. The PARmed-X for pregnancy [21] was administered to ensure readiness to exercise. The study was approved by The University of Western Australia (UWA) Human Ethics Committee (Reference no. RA/4/1/6525) and conducted in accordance with the Declaration of Helsinki. Written informed consent was obtained from all participants.

### Study design

Participants attended a familiarisation session, followed by two experimental trials involving either continuous (CONT) or interval (INTV) stationary cycling performed on separate days in a counterbalanced Latin square design. The three visits were conducted within a 2-week period and were scheduled at least two days apart to minimise any effects from the previous session.

### Experimental procedure

During the familiarisation session, body mass and height were measured. Aerobic fitness was assessed based on the heart rate responses to a modified version of the Aerobic Power Index test [22] on a stationary cycle ergometer (Exertech Ex-10 front access cycle ergometer, Repco Cycle, Huntingdale, Victoria, Australia). This submaximal test has been previously applied in a pregnant population [3, 20]. Briefly, the modified test commenced with cycling at 25 W and increased by 25 W every two minutes until a heart rate equivalent to 75 % of age-predicted maximum (HR_max_) was attained. The use of this sub-maximal exercise protocol based on age-predicted HR_max_ was preferred over conducting a maximal exercise test to avoid subjecting the participants to the discomforts of maximal testing since they were recreational exercisers and did not engage in vigorous exercise in their current pregnancy.

Data from the aerobic test were used to determine the power output that would elicit 65 % HR_max_ for use in the subsequent experimental trials. Following this, participants cycled continuously at the prescribed power output for five to 10 min for the purpose of familiarisation. Next, participants performed two 15-s self-paced higher intensity efforts, with two minutes and 45 s of cycling at the power output equivalent to 65 % HR_max_ in between for recovery. The instruction given for these higher intensity efforts was to “increase the pedalling rate as much as you feel you comfortably can”. The duration of these intervals was based upon pilot work from our laboratory, along with a previous study that utilised higher intensity intervals in a 6-week exercise training intervention for women diagnosed with gestational diabetes [4, 20], and an ongoing study by our laboratory implementing a 14 week interval training program during pregnancy.

### Experimental trials

The subsequent two experimental trials were scheduled for the same time of day to control for circadian variation. At the first trial, participants were required to record all food and drink consumption and to replicate this for the subsequent trial. Quantity of sleep in the prior 24 h was also reported by each participant. Each participant then completed 30 min of cycling on the stationary cycle ergometer commencing and ending with a 5-min warm up and cool down at 30 W. The 20-min conditioning phase between the warm up and cool down consisted of the following performed in a counterbalanced order 1) continuous cycling (CONT) at a steady power output equivalent to 65 % HR_max_ or 2) interval cycling (INTV) consisting of continuous cycling at the same power output as CONT, but with the addition of six 15-s self-paced higher intensity efforts repeated every three minutes, performed with the same instructions as familiarisation. The overall mean duration and intensity of exercise (65 to 75 %HR_max_ and RPE 12–14) for both trials was consistent with recommendations for exercise in pregnancy [1, 23, 24], although it was expected that this threshold would be exceeded for brief periods in response to each self-paced interval.

### Outcome measures

The heart rate response to exercise was monitored continuously throughout the entire trial and recorded at five minute intervals (Polar Heart Rate Monitor, Finland), along with the perceived level of exertion (RPE) using the 6 to 20 Borg Scale [25]. In addition, RPE was recorded after each higher intensity interval and reassessed after one minute of pedalling at the prescribed lower intensity. Mean power output (W) during cycling was measured by a customised computer program interfaced with a stationary cycle ergometer (Cyclemax; School of Sports Science, Exercise and Health, University of Western Australia). In addition, expired air was collected between 20 to 25 min of exercise using a computerised gas analysis system. This consisted of a ventilometer (Universal ventilation meter, VacuMed, Ventura, California USA) that was calibrated prior to each trial as per manufacturer specifications, using a one litre syringe and gas analysers (Ametek Applied Electrochemistry S-3A/1 and CD-3A, AEI Technologies, Pittsburgh, USA) that were calibrated using standard a reference gas of a known physiological concentration. The measured oxygen consumption was used to calculate the energy expenditure [26]. A capillary blood sample (35 μL; Clinitube, Radiometer Medical, Denmark) was taken before and immediately after each exercise trial and analysed for glucose concentrations (ABL™ 700 blood gas system, Radiometer, Copenhagen, Denmark).

The enjoyment of exercise was assessed using the Physical Activity Enjoyment Scale (PACES) [27] immediately following each trial. Briefly, this required participants to rate “how you feel at the moment about the physical activity you have just done” using 18 statements on a 7-point bipolar scale (e.g. “I enjoyed it - I hated it”, “It was very unpleasant - It was very pleasant”). Eleven of 18 items were reverse scored. After reverse scoring items as appropriate, each item was summed to give a total score out of 126, with a higher PACES score reflecting greater levels of enjoyment. In addition, participants provided written responses to the questions “Did you prefer the continuous cycling session or the interval cycling session?” and “why?” based on the two trials that they had performed. Next, they were asked which format of exercise they would like to perform if they were to engage in a 3-month cycling program in pregnancy and were asked to justify their choice.

### Statistical analysis

Heart rate, cycling power output, energy expenditure, oxygen consumption, RPE and PACES (enjoyment) were compared between trials using one-way repeated measures analysis of variance (ANOVA). The blood glucose response to exercise was compared using two-way (time x condition) repeated measure ANOVA. Statistical significance was accepted as a *P* value of ≤ 0.05 (SPSS 20.0 for windows computer software package).

## Results

Sleep duration the night prior was well-matched between trials (*P* = 0.26), with a mean of 7.5 ± 1.2 h of sleep before the CONT trial and 7.3 ± 1.0 h before the INTV trial. Similarly, mean resting heart rate was 89 ± 10 bpm before the CONT and 92 ± 13 bpm before INTV (*P* = 0.29). The characteristics of the exercise trials are displayed in Table 1. Mean power output, heart rate, oxygen consumption and energy expenditure were higher during the 20-min conditioning phase of INTV compared with CONT (Table 1, *P* < 0.05). Of note, the maximum HR reached during the brief self-paced higher intensity intervals was 154 ± 12 bpm [83 ± 6 % age-predicted HR_max_]. Mean blood glucose concentration was similar prior to the start of both trials and decreased in response to exercise (*P* < 0.05), with no difference between conditions.

**Table 1:** Responses to continuous cycling (CONT) compared with interval cycling of equivalent duration (INTV) (mean ± SD)

	CONT	INT
	*n* = 12	*n* = 12
Physiological measures during 20-min conditioning		
Mean power output (W)	78 ± 22	99 ± 18^a^
Mean power output_15 sec effort_ (W)	Not applicable	281 ± 72
Mean heart rate (bpm)	126 ± 8	136 ± 1^a^
Mean percentage HR_max_	68 ± 4	73 ± 5^a^
$\overset{.}{\mathrm{V}}$O_2_ (L.min^−1^)	1.23 ± 0.24	1.57 ± 0.28^a^
Est. energy expenditure (kJ)	506 ± 99	645 ± 116^a^
Glucose_pre_ (mmol∙L^−1^)	5.9 ± 1.0	5.6 ± 1.2
Glucose_post_ (mmol∙L ^-1^)	4.8 ± 0.8^b^	4.6 ± 0.9^b^
Psychological measures during 20-min conditioning		
Mean RPE_overall_	11 ± 2	12 ± 1
Mean RPE_15s_effort_	Not applicable	16 ± 1
Exercise enjoyment (PACES score)	82 ± 21	101 ± 12^a^

Footnotes: *HR*_*max*_ percentage age-predicted heart rate maximum, *PACES* Physical Activity Enjoyment Scale (higher score indicates greater enjoyment of exercise), *RPE* rate of perceived exertion from of 6 to 20 scale; 6 = no exertion, 20 = maximal exertion

^a^denotes significant difference between conditions (*P* < 0.05)

^b^denotes significant difference pre/post within condition (*P* < 0 .05)

Despite the higher overall intensity of INTV compared with CONT, there was no significant difference in mean RPE between conditions. Enjoyment of exercise (PACES score) was higher in INTV compared with CONT (*P* = 0.01; Table 1). From the written responses provided by the participants, all 12 stated that they preferred INTV over CONT. Reasons included that it was more “interesting”, “challenging”, provided a “better workout” and made time “go faster” because the exercise was “broken up”. One participant stated that she had “expected to prefer the continuous cycling” but found that INTV gave her a “sense of accomplishment and better understanding of her exercise capacity”. However, when asked which type of exercise women would prefer if it was a 3-month, thrice weekly cycling program, one participant preferred CONT, eight participants preferred INTV and three preferred a mixture of CONT and INTV.

## Discussion

This study compared the effect of adding brief self-paced higher-intensity intervals to continuous moderate intensity stationary cycling on enjoyment and the physiological responses to exercise in the third trimester of pregnancy. Although interval training of this nature has recently been utilised in women diagnosed with gestational diabetes with favourable effects on maternal fitness and postprandial glucose control, no previous studies have compared the effects of continuous and interval exercise in pregnancy. The addition of six 15-s higher intensity intervals (90 s in total) to 20 min of continuous moderate intensity cycling significantly increased the energy expenditure by 28 %. Importantly, the increased overall exercise intensity with INTV was not associated with a marked difference in RPE. Overall, the session of continuous cycling was perceived as “fairly light” (rating of 11) while the session involving additional brief intervals was perceived as between “fairly light” and “somewhat hard” (rating of 12). This overall RPE rating of INTV indicated a moderate intensity exercise session despite the brief intervals themselves being rated ‘hard’. In addition, exercise enjoyment was higher for INTV compared with CONT, and INTV was the preferred mode of exercise compared with CONT. These findings from a single session of exercise provide a rationale to further examine the effect of incorporating brief self-paced intervals into exercise training for pregnant women which in turn may have implications for exercise prescription.

The greater physiological responses (i.e. heart rate, oxygen consumption and energy expenditure) associated with INTV were expected given that the protocols were not designed to match total work output. The alternative study design of matching total work between CONT and INTV would have required a greater duration of exercise for CONT. Given that a “lack of time” is one of the main barriers to exercise in pregnancy [5], our preference was to match for exercise duration and examine the addition of brief intervals to CONT since the possibility of achieving greater exercise stimulus and energy expenditure within an equivalent amount of time is likely appealing to pregnant women. Of particular interest, the higher overall intensity of INTV was not associated with increased overall perception of effort. This was despite the brief intervals themselves being rated as ‘hard’ in isolation. The overall ‘moderate’ perception of effort with INTV is likely related to the provision of sufficient active recovery at a lower intensity between efforts – an important consideration for ensuring an appropriate overall intensity for the pregnant woman. Furthermore, despite the addition of these brief intervals, INTV was associated with higher ratings of enjoyment based on the PACES. This is consistent with other studies by us and others that have reported higher intensity interval exercise to be more enjoyable than traditional continuous exercise in non-pregnant populations [15, 28]. In the present study, the pregnant women reported INTV as more “interesting” and “challenging” compared with CONT. Since enjoyment of exercise is a major predictor of attendance and adherence to exercise [16], the greater enjoyment associated with INTV may have implications for exercise prescription during pregnancy to promote regular exercise participation. Indeed, when asked which type of exercise women would prefer if it was a 3-month, thrice weekly cycling program, the incorporation of INTV was strongly supported. However, future research is needed to confirm if the findings from a single isolated session of exercise will transfer to regular participation. In addition, it is important to note that enjoyment should not override safety, and so utilisation of this form of exercise training in future studies should include monitoring of maternal and fetal outcomes. Although the fetal responses to the exercise trials were not monitored here, the perceived exertion and heart rate response to the intervals in the present study were consistent with that reported by Halse and colleagues [20] who observed no detrimental effect on obstetric and neonatal outcomes following six weeks of interval exercise training commenced upon diagnosis of gestational diabetes. Furthermore, the addition of six 15-s higher intensity intervals (90 s in total) is of much lesser duration than that maintained by pregnant women during maximal exercise testing [29–31]. However, confirmation of the safety of this mode of exercise training will be needed for programs of longer duration commenced earlier in pregnancy.

A limitation of the current study is the specific focus on healthy women in the third trimester of pregnancy. Thus the findings cannot be generalized to all pregnant women. In addition, the participants included in the present study were recreationally active. It is possible that previously sedentary women may respond differently. With respect to the addition of brief intervals to a regular training program, it is also possible that responses may vary as pregnancy progresses, given the variability in a woman’s emotional and physiological state. Accordingly, this format of exercise needs to be tested on a larger sample of pregnant women of varying exercise habits. In particular, the safety of the women and her fetus must be monitored closely if this format of exercise is performed by previously sedentary women. Nevertheless, this acute study provides the basis for future studies to examine the effects of regular INTV exercise training during pregnancy on maternal and foetal health and wellbeing. Given the many health benefits of regular exercise during pregnancy [1, 10], whether the greater exercise stimulus associated with interval exercise training can benefit the prevention and/or management of certain pregnancy complications such as gestational diabetes also remains to be determined. In the present study, blood glucose concentrations were acutely reduced to a similar extent by both exercise protocols, but since blood glucose was not elevated to start with, future studies in women with impaired glucose tolerance may be warranted.

## Conclusion

In conclusion, the purpose of our study was to investigate the effect of adding brief higher intensity intervals to moderate-intensity continuous cycling on energy expenditure and the enjoyment of exercise in late pregnancy. We have shown that interval cycling, consisting of brief periods of elevated intensity and sufficient recovery at moderate intensity is well tolerated and rated as more enjoyable by women in the third trimester of pregnancy compared with traditional continuous moderate intensity cycling. These findings from a single session of exercise provide a rationale to further examine the effect of incorporating brief self-paced intervals into exercise training for the pregnant woman. However, future research is needed to support the safety and effectiveness of regular performance of this type of training during pregnancy to allow for evidence-based guidelines to inform exercise prescription for the pregnant woman to optimise physiological outcomes as well as enjoyment and long-term adherence to exercise.

## References

[1] Artal R, O’Toole M (2003). Guidelines of the American College of Obstetricians and Gynecologists for exercise during pregnancy and the postpartum period. Br J Sports Med.

[2] Halse RE, Wallman KE, Newnham JP, Guelfi KJ (2013). Pregnant women exercise at a higher intensity during 30 min of self-paced cycling compared with walking during late gestation: implications for 2 h postprandial glucose levels. Metabolism.

[3] Ong M, Guelfi K, Hunter T, Wallman K, Fournier P, Newnham J (2009). Supervised home-based exercise may attenuate the decline of glucose tolerance in obese pregnant women. Diabetes Metab.

[4] Halse RE, Wallman KE, Newnham JP, Guelfi KJ (2014). Home-based exercise training improves capillary glucose profile in women with gestational diabetes. Med Sci Sports Exerc.

[5] Evenson KR, Moos MK, Carrier K, Siega-Riz AM (2009). Perceived barriers to physical activity among pregnant women. Matern Child Health J.

[6] de Jersey SJ, Nicholson JM, Callaway LK, Daniels LA (2013). An observational study of nutrition and physical activity behaviours, knowledge, and advice in pregnancy. BMC Pregnancy Childbirth.

[7] Evenson KR, Wen F (2011). Prevalence and correlates of objectively measured physical activity and sedentary behavior among US pregnant women. Prev Med.

[8] Kardel KR, Johansen B, Voldner N, Iversen PO, Henriksen T (2009). Association between aerobic fitness in late pregnancy and duration of labor in nulliparous women. Acta Obstet Gynecol Scand.

[9] Zavorsky GS, Longo LD (2011). Adding strength training, exercise intensity, and caloric expenditure to exercise guidelines in pregnancy. Obstet Gynecol.

[10] Zavorsky GS, Longo LD (2011). Exercise guidelines in pregnancy. Sports Med.

[11] Penney DS (2008). The effect of vigorous exercise during pregnancy. J Midwifery Womens Health.

[12] Helgerud J, Hoydal K, Wang E, Karlsen T, Berg P, Bjerkaas M, Simonsen T, Helgesen C, Hjorth N, Bach R (2007). Aerobic high-intensity intervals improve VO2max more than moderate training. Med Sci Sports Exerc.

[13] Little JP, Gillen JB, Percival ME, Safdar A, Tarnopolsky MA, Punthakee Z, Jung ME, Gibala MJ (2011). Low-volume high-intensity interval training reduces hyperglycemia and increases muscle mitochondrial capacity in patients with type 2 diabetes. J Appl Physiol.

[14] Wisløff U, Støylen A, Loennechen JP, Bruvold M, Rognmo Ø, Haram PM, Tjønna AE, Helgerud J, Slørdahl SA, Lee SJ (2007). Superior cardiovascular effect of aerobic interval training versus moderate continuous training in heart failure patients a randomized study. Circulation.

[15] Bartlett JD, Close GL, MacLaren DP, Gregson W, Drust B, Morton JP (2011). High-intensity interval running is perceived to be more enjoyable than moderate-intensity continuous exercise: implications for exercise adherence. J Sports Sci.

[16] Ryan R, Frederick C, Lepes D, Rubio N, Sheldon K (1997). Intrinsic motivation and exercise adherence. Int J Sport Psychol.

[17] Mudd LM, Owe KM, Mottola MF, Pivarnik JM (2013). Health benefits of physical activity during pregnancy: an international perspective. Med Sci Sports Exerc.

[18] Arem H, Moore SC, Patel A, Hartge P, de Gonzalez AB, Visvanathan K, Campbell PT, Freedman M, Weiderpass E, Adami HO (2015). Leisure time physical activity and mortality: a detailed pooled analysis of the dose–response relationship. JAMA Int Med.

[19] Wen CP, Wai JPM, Tsai MK, Yang YC, Cheng TYD, Lee M-C, Chan HT, Tsao CK, Tsai SP, Wu X (2011). Minimum amount of physical activity for reduced mortality and extended life expectancy: a prospective cohort study. Lancet.

[20] Halse RE, Wallman KE, Dimmock JA, Newnham JP, Guelfi KJ. Home-Based Exercise Improves Fitness and Exercise Attitude and Intention in GDM Women. Med Sci Sports Exerc. 2015;47(8):1698-704. 10.1249/MSS.0000000000000587 25412300

[21] Wolfe L, Mottola M (2002). PARmed-X for pregnancy.

[22] Telford R, Minikin B, Hahn A, Hooper L (1989). A simple method for the assessment of general fitness: the tri-level profile. Aust J Sci Med Sport.

[23] Royal College of Obstetricians and Gynaecologists (2006). Exercise in pregnancy. RCOG statement 4.

[24] Davies GA, Wolfe LA, Mottola MF, MacKinnon C (2003). Joint SOGC/CSEP clinical practice guideline: exercise in pregnancy and the postpartum period. Can J Appl Physiol.

[25] Borg GA (1982). Psychophysical bases of perceived exertion. Med Sci Sports Exerc.

[26] McArdle WD, Katch FI, Katch, VL. Exercise physiology: Energy, nutrition, and human performance. Philadelphia : Wolters Kluwer/Lippincott Williams & Wilkins Health; 2010.

[27] Kendzierski D, DeCarlo KJ. Physical Activity Enjoyment Scale: Two validation studies. J Sport Exerc Psychol. 1991;13(1):50-64.

[28] Crisp NA, Fournier PA, Licari MK, Braham R, Guelfi KJ (2012). Adding sprints to continuous exercise at the intensity that maximises fat oxidation: Implications for acute energy balance and enjoyment. Metabolism.

[29] MacPhail A, Davies GA, Victory R, Wolfe LA (2000). Maximal exercise testing in late gestation: fetal responses. Obstet Gynecol.

[30] Lotgering FK, van Doorn MB, Struijk PC, Pool J, Wallenburg HC (1991). Maximal aerobic exercise in pregnant women: heart rate, O2 consumption, CO2 production, and ventilation. J Appl Physiol.

[31] Sady S, Carpenter M, Sady M, Haydon B, Hoegsberg B, Cullinane E, Thompson P, Coustan D (1988). Prediction of VO2max during cycle exercise in pregnant women. J Appl Physiol.

